# Presentation and Outcomes of Patients With End-Stage Kidney Disease Hospitalized With COVID-19 at a Tertiary Center in Riyadh, Kingdom of Saudi Arabia

**DOI:** 10.7759/cureus.23575

**Published:** 2022-03-28

**Authors:** Mohammed Tawhari, Eythar Alrushid, Ghadah Alquwaiee, Shuq Alanazi, Joud Alkhudair, Abdulaziz Aldalaan, Shikah Alsuwaid, Aljawharah Alabdulkarim, Fawaz Tawhari, Khaled M Hattan, Ibrahim Tawhari, Mansoor Radwi

**Affiliations:** 1 Nephrology, King Abdulaziz Medical City Riyadh, Riyadh, SAU; 2 Nephrology, King Abdullah International Medical Research Center, Riyadh, SAU; 3 Nephrology, King Saud Bin Abdulaziz University for Health Sciences, College of Medicine, Riyadh, SAU; 4 Medicine and Surgery, King Saud Bin Abdulaziz University for Health Sciences, College of Medicine, Riyadh, SAU; 5 Pharmacy, Armed Forces Hospital, Khamis Mushait, SAU; 6 Medicine and Surgery, Jazan University, Jazan, SAU; 7 Medicine, King Khalid University, Abha, SAU; 8 Internal Medicine and Hematology, University of Jeddah, College of Medicine, Jeddah, SAU

**Keywords:** morbidity and mortality, severity, predictive factor, sars-cov-2 (severe acute respiratory syndrome coronavirus -2), end stage kidney disease (eskd), covid 19

## Abstract

Background

Patients with end-stage kidney disease (ESKD) are disproportionately vulnerable to COVID-19 and its complications due to the older age and significant burden of comorbid conditions. Data about the impact of COVID-19 on the ESKD population in the Kingdom of Saudi Arabia is scarce, and this study aims to bridge this gap.

Method

This is a retrospective cohort study that included ESKD patients who were receiving either in-center hemodialysis (HD) or peritoneal dialysis (PD) for at least three months and were hospitalized due to COVID-19 at King Abdulaziz Medical City in Riyadh (KAMC) between March 2020 and March 2021. Of note, the in-center hemodialysis means that the patients come to the dialysis center three times per week to receive their dialysis sessions, as home hemodialysis is not available at our center. Multivariate logistic regression was performed to explore the association of clinical characteristics and laboratory parameters with ICU admission and mortality.

Results

A total of 104 patients were included in the analysis. The mean age was 62.6 (SD=17.4) years, 101 (97%) were on HD, predominantly through a central venous catheter (72%), and 53 patients (51%) were male. Patients with COVID-19 were either asymptomatic (42%) or had mild symptoms (37%), mainly cough and fever. At the time of admission, 37 patients (36%) had extrapulmonary symptoms, and 13 patients (12%) had altered mental status. Normal chest X-ray (48%), followed by bilateral lung infiltrates (24%), and unilateral lung infiltrate (11%) were the most common radiological findings. We did not observe any thromboembolic events. Twenty patients (19%) required ICU admission and 19 patients (18%) died during hospitalization. Predictors for in-hospital mortality were: 1) the need for inotropes (adjusted OR: 53.01, p=0.006), 2) age (adjusted OR: 1.07, p=0.019), and 3) C-reactive protein (CRP) level on admission (adjusted OR: 1.02, p=0.04). We did not find any strong predictor for ICU admission.

Conclusion

Our study demonstrated that COVID-19 carries significant mortality and morbidity in the ESKD population. Age, inotropic support requirement and elevated CRP on admission predicted mortality in our population. The high rate of adverse outcomes of COVID-19 among ESRD patients calls for strict implementation of preventive measures, including vaccination, social distancing, and universal masking at the level of both the healthcare providers and patients. Further studies are needed to assess the association of COVID-19 and hypercoagulability ESKD population.

## Introduction

Coronavirus disease 2019 (COVID-19) is a respiratory infection caused by a novel coronavirus, severe acute respiratory syndrome coronavirus 2 (SARS-CoV-2). It was initially detected in China in December 2019, subsequently spread globally, and was declared a pandemic disease by World Health Organization (WHO) on March 11, 2020 [1,2].

Individuals with COVID-19 can present with no symptoms (i.e., asymptomatic infection), mild upper respiratory symptoms, or severe bilateral viral pneumonia resulting in respiratory failure requiring advanced respiratory support and death [3,4]. Risk factors for poor outcomes in patients with COVID-19 include advanced age, male gender, obesity, comorbidities (e.g., diabetes mellitus, hypertension, cardiovascular diseases, chronic kidney disease, chronic lung diseases, immunosuppression, sickle cell anemia, moderate-to-severe asthma, pregnancy, and cancer) [5-7].

End-stage kidney disease (ESKD) is a major risk factor that is associated with unfavorable outcomes in COVID-19. ESKD patients on hemodialysis (HD) require special care during the COVID-19 pandemic, as they are considered relatively immunocompromised, have multiple comorbidities, and are often in close proximity to other patients during hemodialysis. In the Kingdom of Saudi Arabia, the ESKD prevalence and incidence of ESKD have increased over the past three decades [8]. The prevalence of patients with ESKD on chronic dialysis is 513 cases per million population, while the annual incidence of patients with ESKD is 136 cases per million population [8].

Data regarding clinical presentation and outcomes of COVID-19 in patients with ESKD in Saudi Arabia is limited. Information regarding clinical presentation, prognostic factors, the proportion of patients who required hospitalization or ICU admission, and mortality rate are not well reported. Our study aims to fill this gap by presenting a detailed clinical presentation, management, and outcomes of COVID-19 in dialysis patients who presented to a tertiary care hospital in Riyadh, Saudi Arabia.

## Materials and methods

We conducted a retrospective cohort study at King Abdulaziz Medical City (KAMC), Riyadh, Saudi Arabia. KAMC is a tertiary hospital and is part of government-funded multispecialty health in Saudi Arabia. The data was collected after obtaining the IRB approval from King Abdullah International Medical Research Center (NRC21R/150/04).

We included all patients who were 18 years of age or older with ESKD, who had been receiving hemodialysis at KAMC dialysis center or peritoneal dialysis for at least three months and were diagnosed with COVID-19 between March 2, 2020, and March 31, 2021. The diagnosis of COVID-19 was based on nasopharyngeal swab positivity for SARS-CoV-2 by polymerase chain reaction (PCR). ESKD patients with functioning allografts and those who had been on dialysis for less than three months were excluded.

We reviewed the electronic medical record system "Best care", and the following variables were collected in a spreadsheet: age, gender, body mass index (BMI), smoking history, diabetes mellitus (DM), hypertension, dyslipidemia, history of lung diseases, stroke, cancer, coronary artery disease, past history of congestive hear failure (CHF), past history of deep vein thrombosis (DVT), etiology of renal failure, dialysis access, mode of dialysis, date of COVID-19 admission, date of the positive test, clinical status on admission, extrapulmonary symptoms, altered level of consciousness, chest X-ray (CXR) findings, oxygen requirement on admission, serum albumin level, erythrocyte sedimentation rate (ESR) level, C-reactive protein (CRP) level, ferritin level, D-dimer value, troponin level, white blood cell (WBC) count on admission, highest white blood cell count, lowest WBC reading, lowest lymphocyte count, hemoglobin level before admission, intensive care unit admission (ICU), oxygen requirement in ICU, need for mechanical ventilation, need for non-invasive positive pressure ventilation (NIPPV), need for high-flow nasal canula (HFNC), DVT events during hospitalization, DVT prophylaxis, antibiotics use, hydroxychloroquine use, azithromycin use, tocilizumab use, remdisivir use, favipiravir use, renal replacement therapy mode in hospital, need for inotropes, use of erythropoietin, use of intravenous iron supplementation, use of convalescent plasma, positive blood cultures, condition on discharge, date of death and cause of death. Of note, asymptomatic individuals were defined as those who tested positive for SARS-CoV-2 without reporting any symptoms. Our center performs active surveillance testing for anyone who comes in direct contact with a positive/suspected subject and before any planned admission for any procedure, for example, a dialysis catheter insertion or arteriovenous fistula creation. If the patient tests positive for SARS-CoV-2, then the patient will be admitted to the hospital to receive hemodialysis in an isolation room to prevent the spread of the infection to other dialysis patients in the center. The isolation is usually discontinued after the completion of a total of 10-14 days from the positive test, and then the patient can be discharged to receive their dialysis at the center and can proceed with any planned procedure. 

Categorical variables were presented as frequencies and percentages, whereas continuous variables were presented as mean ± standard deviation. Multivariate logistic regression was performed to explore the association of clinical characteristics and laboratory parameters with ICU admission and mortality. Variable selection was performed using AIC (Akaike information criteria)-based backward stepwise selection. The p-values then validated the final models for Hosmer-Lemeshow goodness of fit and likelihood ratio tests.

## Results

The baseline characteristics of our patients are shown in Table 1.

**Table 1:** Baseline characteristics VTE - venous thromboembolism; DM - diabetes mellitus; HTN - hypertension; GN - glomerulonephritis; PD - peritoneal dialysis; EF - ejection fraction; unknown EF - no echocardiogram was available in the patient's file; ESKD - end-stage kidney disease; AV - arteriovenous

Variable	N (SD)	%
Age in years	62.6 (17.4)	
Gender (male)	53	51%
Body-mass index kg/m^2^	29.2 (7.7)	
History of smoking		
Current smoker	5	5%
Ex-smoker	5	5%
Diabetes mellitus	82	79%
Hypertension	101	97%
Dyslipidemia	41	39%
Lung disease	14	13%
Stroke	14	13%
Cancer	7	7%
Coronary artery disease	29	28%
Heart failure with reduced EF		
Yes	57	55%
No	27	26%
Unknown EF	20	19%
History of VTE	13	12%
Cause of ESKD		
DM	52	50%
HTN	9	9%
GN	5	5%
Hereditary	3	3%
Others	35	34%
Previous transplant	9	9%
Dialysis access		
Tunneled catheter	75	72%
AV fistula/graft	26	25%
PD	3	3%

We identified a total of 104 patients who met our inclusion criteria. The mean age was 62.6 (SD=17.4) years, and the average BMI was 29.2 (SD=7.7) kg/m². Fifty-three patients (51%) were male. The most common comorbidities were hypertension (97%), diabetes (79%), dyslipidemia (39%), coronary artery disease (28%), lung disease (13%), stroke (13%), and cancer (7%).

Most of our cohort had normal left ventricular ejection fraction (55%), 27 patients (26%) had heart failure with reduced ejection fraction, and 20 patients (19%) did not have echocardiogram results in their files. Thirteen patients (12%) had a previous history of venous thrombosis: seven patients (7%) had lower extremities DVT, five patients (5%) had upper extremities DVT, and only one patient (1%) had a pulmonary embolism (PE). The most common identifiable causes of ESKD in our cohort were diabetes (50%), followed by hypertension (9%), glomerulonephritis (5%), and hereditary (3%). Over one-third (34%) of our cohort had ESKD from other causes, such as obstruction, interstitial nephritis, or unknown causes. Of note, only nine patients (9%) had failed renal allografts.

In terms of dialysis access, most of our cohort (72%) were receiving hemodialysis via a tunneled central venous catheter (CVC), followed by arteriovenous (AV) fistula/graft (25%), and only three (3%) patients were on peritoneal dialysis (PD).

Clinical features and outcomes of COVID-19 infection are shown in Table 2.

**Table 2:** Clinical features and outcomes of COVID-19 NIPPV - non-invasive positive pressure ventilation; MV - mechanical ventilation; CXR - chest X-ray; VTE - venous thromboembolism; IV - Intravenous; RRT - renal replacement therapy

Variable	N	%
Clinical status on admission		
Asymptomatic	44	42%
Mild symptoms without supplemental O_2_	39	37%
Symptomatic requiring supplemental O_2_	16	15%
Requiring NIPPV or MV	5	5%
Extrapulmonary symptoms	37	36%
Altered level of consciousness	13	12%
CXR finding		
Normal	50	48%
Bilateral infiltrate	25	24%
Unilateral infiltrate	12	11%
Pulmonary edema	10	10%
Pleural effusion	6	6%
Others	1	17%
Intensive care admission	20	19%
Mechanical ventilation	15	14%
NIPPV	18	17%
High flow nasal cannula	13	12%
VTE during admission	0	0%
VTE prophylaxis	80	77%
Antibiotics use	73	70%
Steroid use	41	39%
Medications:		
Hydroxychloroquine	2	2%
Azithromycin	17	16%
Tocilizumab	7	7%
Inotropic support	13	12%
Convalescent plasma	5	5%
Erythropoietin	50	48%
IV iron	12	11%
RRT in hospital		
Hemodialysis	102	98%
Blood culture positivity:		
Gram-positive	18	17%
Gram-negative	3	3%
Fungal	3	3%
Outcome of hospitalization		
Independent	79	76%
Requires aids	6	6%
Death	19	18%
Cause of death		
COVID-19	16	15%
Other cause	3	3%

The laboratory parameters of our cohort are presented in Table 3.

**Table 3:** Laboratory findings CRP - C-reactive protein; ESR - erythrocyte sedimentation rate; WBCs - white blood cell count; Trop I - troponin I

Variable	Mean (standard deviation)	Normal value
Hemoglobin (g/l)	112.8 (19.8)	120-160 g/l
Albumin (g/l)	29.2 (6.3)	35-52 g/l
CRP (mg/l)	116.4 (79.5)	< 8 mg/l
ESR (mm/hr)	81.5 (33.8)	0-30 mm/hr
Lowest lymphocyte count (cell/l)	0.8 (0.9) x10^9^/l	0-0.1 x10^9^/l
Lowest WBCs (cell/l)	4.2 (1.8) x10^9^/l	4-11 x10^9^/l
Highest WBCs count (cell/l)	14.9 (10.2) x10^9^/l	4-11 x10^9^/l
Ferritin level on admission (ug/l)	1973.7 (3823.6)	21.8-274.6 ug/l
D-dimer on admission (mg/l)	2.2 (3)	<0.5 mg/l
Trop I on admission (pg/ml)	163 (422.3)	<15.6 pg/ml for females; <34.2 pg/ml for males

Upon admission, 44 patients (42%) were asymptomatic, 39 patients (37%) had mild symptoms without needing supplemental oxygen, 16 patients (15%) required supplemental oxygen, and five patients (5%) required respiratory support through non-invasive positive pressure ventilation (NIPVV) or mechanical ventilation. Thirty-seven patients (36%) presented with extrapulmonary symptoms, including headache, sore throat, GI symptoms, and malaise. Altered mental status was present in 13 patients (12%). Only 77% of patients received DVT prophylaxis during their hospitalization. Given the observational nature of the study, we were not able to identify the reason for not having DVT prophylaxis in 23% of the cohort. Only 24 patients (23%) had positive blood cultures, with gram-positive organisms being the commonest (17%), followed by gram-negative organisms (3%) and fungi (3%). The most common sources of the infections were line-related in 13 patients (12%), pneumonia in eight patients (8%), and skin/soft tissue infection in three patients (3%).

The most common radiological manifestation was normal chest X-ray (48%), followed by bilateral lung infiltrate (24%), followed by unilateral lung infiltrate (11%), pulmonary edema (10%), and pleural effusion (6%). The computerized tomography (CT) scans were performed for four patients (4%), and all of them showed bilateral ground-glass opacification with pleural effusions.

Regarding treatments used during hospitalization, antibiotics were administered to 70% of our cohort, and 39% of them received glucocorticoids. Inotropic support was required for 12% of our cohort. Other administered medications include erythropoietin (48%), intravenous iron (11%), azithromycin (16%), and hydroxychloroquine (2%). Tocilizumab and convalescent plasma were administered to 7% and 5%, respectively. During hospitalization, hemodialysis was the most common modality of renal replacement therapy (98%), with only two patients who were maintained on peritoneal dialysis.

During hospitalization, intensive care admission was required for 20 patients (19%), with 15 patients (14%) required mechanical ventilation, 18 patients (17%) required NIPPV, and 13 patients (12%) required high flow nasal cannula. None of our patients developed a thromboembolic event.

At the time of discharge, 79 patients (76%) of our cohort were independent, meaning that they were able to ambulate without walking aids or assistance from another person. Six patients (6%) required physical aids such as walking frames, wheelchairs, or bathroom aids. Nineteen patients (18%) died during the hospitalization, 16 of them were attributed to COVID-19, and three deaths were determined to be unrelated to COVID-19. Out of the three deaths, two were due to gram-negative sepsis, and one was due to cardiac arrhythmia. Of note, all deaths occurred during ICU stay.

The subgroup analysis showed that patients who had COVID-19 symptoms at the time of admission were more likely to be admitted to ICU when compared to asymptomatic patients (28% vs. 7%, p<0.01). In addition, the symptomatic group had a higher mortality rate when compared to the asymptomatic group (23% vs. 11%, p=0.1); however, this did not reach statistical significance.

In multivariate logistic regression analysis (Table 4), the predictors of mortality among our patients were the need for inotropes (adjusted OR: 53.01, 95% CI: 3.18-83.37, p=0.006), age (adjusted OR: 1.07, 95% CI: 1.01-1.1, p=0.019), CRP level on admission (adjusted OR: 1.02, 95% CI: 1.00-1.05, p=0.04), lowest total WBCs (adjusted OR: 1.47, 95% CI: 0.93-2.33, p=0.1), and being a current smoker (adjusted OR: 3.45, 95% CI: 1.08-10.99, p=0.05).

**Table 4:** Predictors of mortality in ESRD patients with COVID-19 ESRD - end-stage kidney disease; CRP - C-reactive protein; WBCs - white blood cell count

Variable	Crude OR (95 % CI)	Adjusted OR (95 % CI)	P-value
Age	1.06 (1.02,1.11)	1.07 (1.01,1.18)	0.019
Need of vasopressors	32.86 (7.29,48.19)	53.01 (3.18,83.37)	0.006
CRP on admission	1.02 (1.01,1.03)	1.02 (1.00,1.05)	0.046
Lowest total WBCs	1.54 (1.14,2.08)	1.47 (0.93,2.33)	0.097
Smoking (current)	3.83 (1.3,48.93)	3.45 (1.08,10.99)	0.05

The predictors of ICU admission (Table 5) were age (adjusted OR: 1.05, 95% CI: 1.00-1.1, p=0.05), requirement of supplemental O_2_ at the time of admission (adjusted OR: 4.46, 95% CI: 0.49-21.1, p=0.05), BMI of 35 or more (adjusted OR: 4.61, 95% CI: 0.84-25.18, p=0.08) and being a female (adjusted OR: 2.92, 95% CI: 0.57-14.91, p=0.19).

**Table 5:** Predictors of ICU admission in ESRD patients with COVID-19 ESRD - end-stage kidney disease; BMI - body mass index

Variable	Crude OR (95 % CI)	Adjusted OR (95 % CI)	P-value
Age	1.05 (1.01,1.09)	1.05 (1.00,1.1)	0.05
Requirement of supplemental oxygen on admission	4.67 (1.57,13.89)	4.46 (1.49,21.14)	0.05
BMI >35	5.01 (1.51,16.63)	4.61 (0.84,25.18)	0.078
Gender (female)	2.18 (0.74,6.44)	2.92 (0.57,14.91)	0.197

## Discussion

ESKD population is disproportionately vulnerable to COVID-19 complications [9,10] as they are immunocompromised [11] and have a large burden of comorbid conditions. In Saudi Arabia, the majority of ESKD patients are receiving in-center hemodialysis, and hence it is prudent to understand the effect of the global pandemic on our ESKD population. In this study, we explored the presentation and outcomes of COVID-19 among ESKD receiving peritoneal or in-center hemodialysis. 

We closely examined the clinical course of 104 patients; three of them were on PD, admitted with COVID-19 between March 2020 and March 2021. During that period, it was our practice to admit any in-center hemodialysis patient who tested positive for SARS-CoV-2, regardless of the severity of symptoms, to maintain them on dialysis in an isolated setting that was not possible at the dialysis center. This explains our finding that 79% of our patients were either asymptomatic or had mild symptoms that did not need supplemental oxygen. The observation that only three of those who were admitted were on PD can be explained by the ability of PD patients to maintain their dialysis while they self-isolate at home unless they have moderate or severe COVID-19 symptoms that require hospitalization. It should be noted that 42% of our patients were totally asymptomatic and were admitted just to receive hemodialysis, and this can affect the generalizability of our results.

The mortality rate among our ESKD cohort reached 18%. However, the subgroup analysis showed that the mortality rate was 23% in patients who had COVID-19 symptoms at the time of admission. Our literature search showed a 20-50% mortality rate among ESKD patients with COVID-19 [12-15]. This study's marginally lower mortality rate can be attributed to having a large proportion of patients with mild disease. It should be noted that studies that reported COVID-19 outcomes in ESKD included around 40% of asymptomatic or mildly symptomatic patients, which is a much lower proportion when compared to 79% in our study [12,13]. The mortality rate of 11% among the patients with asymptomatic presentation could mean that some of them developed symptoms and became sicker during the course of their hospitalization. Like the previous reports, the patients who died were older [5-7,13,15-17], had a higher CRP level [13,18], and required vasopressors or ICU admission [13]. Another note that is worth mentioning is that not all the in-hospital deaths were directly attributable to COVID-19, which may indicate that even if ESKD patients recover from COVID-19, they are still at risk of dying from other causes [18]. The number of deaths from other causes was very small, making it difficult to draw any conclusion about whether COVID-19 may have had a role in the occurrence of these complications that led to death.

Earlier reports suggested that the ESKD population with COVID-19 presents atypical symptoms, e.g., gastrointestinal symptoms being the most dominant in such population [19]. However, our results are consistent with the more recent reports that demonstrated COVID-19 presentation in the ESKD population are similar to their general population counterparts with COVID-19 (i.e., respiratory symptoms of fever, cough, and shortness of breath being the commonest presenting symptoms) [10,13,20,21]. We also observed that respiratory symptoms did not occur in isolation, and over a third of our patients had extrapulmonary symptoms, mainly gastrointestinal distress. Altered mental status was present in 12.5%, which is also consistent with previous reports [20]. The most common X-ray abnormalities were lung infiltrates, either bilateral or unilateral, which is consistent with previous studies and similar to the general population with COVID-19 [13,22]. Since only a small number of our patients had CT scans, we were unable to make any conclusion regarding the CT scan findings. 

We want to address a few paradoxical observations that conflict with what has been reported in the literature. First, none of our patients developed any thrombotic events, despite the fact that over 20% of them did not receive pharmacological prophylaxis. While earlier reports suggested that COVID-19 is associated with an increased incidence of VTE, particularly among acutely ill patients [23,24], there are potential explanations for our finding. First, most of our patients were asymptomatic or had minimal symptoms, i.e., were not sick enough to become hypercoagulable. Second, the hemodialysis patients, who constitute most of our patients, receive heparin with each hemodialysis session, which could have offered some protection. Given the observational nature of our study and the small sample size, we cannot conclude that hemodialysis patients are at lower risk of VTE and still advocate that hemodialysis patients hospitalized for COVID-19 require prophylactic measures against VTE unless contraindicated. Of note, although we were unable to identify the reasons for not receiving the DVT prophylaxis in over 20% of our patients, this is unlikely to have affected our finding of no DVT. However, further audits are needed to address the importance of DVT prophylaxis at our center. 

Second, we found no significant association between leukopenia and mortality, unlike previous reports on the general population [25]. However, previous reports did not find any significant association between lymphopenia and disease severity when focusing on immunocompromised patients [26]. So, it is plausible that the inflammatory markers lose their predictive value in the immunocompromised population, or alternatively, the lack of statistical detection was because the majority of our cohort had a mild disease or were asymptomatic.

Third, high BMI was not significantly associated with mortality nor with critical care admission, despite the previously documented association among the general population [5-7,27]. We reiterate that our cohort is different, and thus findings from previous studies are likely not to be reproduced here. Further studies are needed to shed light on the impact of obesity and the degree of leukopenia on COVID-19 infection among the ESKD population, especially those who have mild symptoms.

Lastly, the use of erythropoietin (EPO) in the setting of COVID-19 has been controversial, and one should balance the risk of increasing thrombosis risk against the immunomodulatory effects of EPO [28,29]. However, our study showed that almost half of our cohort received EPO and yet no VTE event. While this is reassuring, this issue is better addressed through well-designed randomized controlled studies.

Our study has strengths and limitations. The main strength of our study is that it described the presentation and outcomes of COVID-19 in one of the largest tertiary care centers in Saudi Arabia. In addition, the presentation and outcomes of COVID-19 among our dialysis population are consistent with what has been reported in international reports. The study also generated areas for further research, particularly the risk of DVT among ESKD patients with COVID-19.

The limitations of our study are the observational nature, being limited to a single center, and the relatively small sample size. In addition, the study only evaluated the in-hospital course and did not have a post-discharge follow-up. It should be noted that only a small number of our patients received medications besides corticosteroids and antibiotics, which limited our ability to make any conclusion about the effectiveness of these medications, and made it difficult to decide if the mortality rate changed in the latter half of the year, as the knowledge about the COVID-19 improved. Also, the asymptomatic presentation of the majority of our cohort limits the generalizability of our results to patients presenting with more severe symptoms and may have skewed our results.

## Conclusions

Our study demonstrated that COVID-19 carries significant mortality and morbidity in the ESKD population. Age, inotropic support requirement, and elevated CRP on admission predicted mortality in our population. The high rate of adverse outcomes of COVID-19 among ESRD patients calls for strict implementation of preventive measures, including vaccination, social distancing, and universal masking at the level of both the healthcare providers and patients. Further studies are needed to assess the association between COVID-19 and the hypercoagulability of the ESKD population. 
